# Blood pressure trajectories over 35 years and dementia risk: A retrospective study: The HUNT study

**DOI:** 10.3389/fnagi.2022.931715

**Published:** 2022-09-15

**Authors:** Geir Selbaek, Josephine Stuebs, Knut Engedal, Vladimir Hachinski, Knut Hestad, Cathrine Selnes Trevino, Håvard Skjellegrind, Yehani Wedatilake, Bjørn Heine Strand

**Affiliations:** ^1^Norwegian National Centre for Aging and Health, Vestfold Hospital Trust, Tønsberg, Norway; ^2^Department of Geriatric Medicine, Oslo University Hospital, Oslo, Norway; ^3^Faculty of Medicine, Institute of Clinical Medicine, University of Oslo, Oslo, Norway; ^4^Department of Clinical Neurological Sciences, Robarts Research Institute, University of Western Ontario, London, ON, Canada; ^5^Department of Research, Innlandet Hospital Trust, Brumunddal, Norway; ^6^Department of Health and Nursing Science, Faculty of Health and Social Sciences, Inland Norway University of Applied Sciences, Elverum, Norway; ^7^Department of Public Health and Nursing, Faculty of Medicine and Health Sciences, HUNT Research Centre, Norwegian University of Science and Technology, Levanger, Norway; ^8^Levanger Hospital, Nord-Trøndelag Hospital Trust, Levanger, Norway; ^9^The Research Centre for Age-Related Functional Decline and Disease, Innlandet Hospital Trust, Ottestad, Norway; ^10^Department of Physical Health and Ageing, Norwegian Institute of Public Health, Oslo, Norway

**Keywords:** blood pressure, dementia, trajectory, cohort study, Alzheimer, vascular dementia

## Abstract

High blood pressure is a well-established risk factor of dementia. However, the timing of the risk remains controversial. The aim of the present study was to compare trajectories of systolic blood pressure (SBP) over a 35-year follow-up period in the Health Survey in Trøndelag (HUNT) from study wave 1 to 4 in people with and without a dementia diagnosis at wave 4 (HUNT4). This is a retrospective cohort study of participants aged ≥ 70 years in HUNT4, where 9,720 participants were assessed for dementia. In the HUNT study all residents aged ≥ 20 years have been invited to four surveys: HUNT1 1984–86, HUNT2 1995–97, HUNT3 2006–08 and HUNT4 2017–19. The study sample was aged 70–102 years (mean 77.6, *SD* 6.0) at HUNT4, 54% were women and 15.5% had dementia, 8.8% had Alzheimer’s disease (AD), 1.6% had vascular dementia (VaD) and 5.1% had other types of dementia. Compared to those without dementia at HUNT4, those with dementia at HUNT4 had higher SBP at HUNT1 and HUNT2, but lower SBP at HUNT4. These differences at HUNT1 and 2 were especially pronounced among women. Results did not differ across birth cohorts. For dementia subtypes at HUNT4, the VaD group had a higher SBP than the AD group at HUNT2 and 3. Age trajectories in SBP showed that the dementia group experienced a steady increase in SBP until 65 years of age and a decrease from 70 to 90 years. SBP in the no- dementia group increased until 80 years before it leveled off from 80 to 90 years. The present study confirms findings of higher midlife SBP and lower late-life SBP in people with dementia. This pattern may have several explanations and it highlights the need for close monitoring of BP treatment in older adults, with frequent reappraisal of treatment needs.

## Introduction

Dementia is a chronic, progressive syndrome which affects cognition, behavior, and daily life functioning. The steeply rising prevalence of dementia presents an immense individual, societal and economic burden. In 2019, there were 57,4 million persons with dementia globally. This number is projected to increase to 152,8 million by 2050 (GBD Dementia Forecasting Collaborators, 2022). In Norway, 101,000 persons have dementia, set to exceed 236,000 by 2050 (Gjøra et al., 2021). With a few exceptions, such as antiretroviral therapy for HIV-associated dementia, treatment of vascular diseases causing dementia and the recently FDA approved monoclonal antibody, aducanumab, there is at present no disease-modifying treatment available for any of the diseases causing dementia. However, modifiable lifestyle risk factors for dementia may be a target for intervention. The recent Lancet commission on dementia prevention, intervention and care identified 12 modifiable risk factors and estimated that more than 40% of dementia cases could be delayed or prevented by excluding these risk factors (Livingston et al., 2020).

Hypertension is one of these risk factors. It has been suggested that the observed decrease in dementia incidence over the last decades is partly due to better blood pressure (BP) control, especially in people with midlife hypertension (Satizabal et al., 2016). Ample evidence suggests that midlife hypertension is associated with an increased risk of dementia (McGrath et al., 2017; Abell et al., 2018). However, as age increases the association is attenuated and might even be reversed. Previous studies have found that hypotension in old age may be associated with an increased risk of dementia (Hestad et al., 2005; Qiu et al., 2009; Gabin et al., 2017). Alternatively, both late-life hypertension and hypotension may be associated with an increased risk of dementia (Walker et al., 2019). This may be due to reverse causality. The development of degenerative brain disorder may induce a decrease in BP. This is particularly relevant for the most common dementia disorder, Alzheimer’s disease (AD), in which the degenerative process starts in the brain decades before cognitive and functional impairment become apparent (Frisoni et al., 2022). The association between hypertension and risk of AD is still not well understood. Whereas there seems to be a rather robust association between midlife diastolic hypertension and AD risk, the results regarding midlife systolic hypertension and AD risk are conflicting (Walker et al., 2017). Few studies have investigated the association between midlife hypertension and risk of vascular dementia (VaD) but the association between hypertension and risk of VaD seems to be more robust than for AD (Walker et al., 2017). Sex differences in the association between hypertension and dementia risk are not well characterized and a recent review concluded that studies rarely, and inconsistently analyzed or reported sex effects (Blanken and Nation, 2020). Two recent studies indicated that sex differences exist both for risk of dementia (Gong et al., 2021) and the risk of memory decline (Anstey et al., 2021).

To be able to intervene in clinical settings, we need precise information on patterns of how risk factors change over the life course. However, very few studies have been able to follow the trajectories of blood pressure from early or midlife until late life. A recent review identified only four trajectory studies reporting on risk of all-cause dementia and three studies reporting on risk of AD or VaD. Only two of the studies had a follow-up longer than 10 years (Peters et al., 2020).

The present study aims to test the hypothesis that midlife hypertension is associated with dementia in late life, but that this association is attenuated and even reversed with increasing age. Furthermore, we hypothesize that different risk profiles exist between men and women, older and younger age groups, and between participants with AD and VaD.

## Materials and methods

### Study population

In this retrospective cohort study, we employed data from the Trøndelag Health (HUNT) study for our analyses. The HUNT study is a unique database of questionnaire data, clinical measurements, and biological samples from the former Nord-Trøndelag county’s population from 1984 onward. The study includes data from persons 20 years or older, gathered during four waves: HUNT1 (1984–1986), HUNT2 (1995–1997), HUNT3 (2006–2008), and HUNT4 (2017–2019). In each HUNT wave, data were collected over a 2-year period (Åsvold et al., 2022). In HUNT4 all participants who were 70 years and older were invited to participate in the HUNT4 70+ study (Gjøra et al., 2021) where they underwent cognitive assessments.

Our study population included participants from the HUNT4 70+ study, born 1914–49. Among a total of 9,904 participants, those with missing dementia assessment at HUNT4 (*n* = 178) and/or no BP measurements in any waves, HUNT1-HUNT4 (*n* = 6) were excluded. A total of 9,720 individuals were included in the analysis. In this study population of HUNT4-participants, we studied systolic blood pressure (SBP) trajectories retrospectively during the HUNT1-HUNT4 waves and analyzed them by dementia status at HUNT4.

### Procedures for diagnosis of dementia or mild cognitive impairment

In HUNT4 70+, dementia diagnoses were set by experts from a diagnostic group of nine medical doctors with both scientific and clinical expertise (geriatrics, neurology or old-age psychiatry). A diagnosis was made for each case by two experts independently, applying the DSM-5 diagnostic criteria to classify the following conditions: no cognitive impairment, mild cognitive impairment (mild neurocognitive disorder), dementia (major neurocognitive disorder) and dementia subtypes; AD, VaD, Lewy body dementias (LBD), frontotemporal dementia (FTD), mixed dementia, other specified dementia and unspecified dementia (Gjøra et al., 2021). If no consensus for the diagnosis was reached a third expert was consulted. During the diagnostic process the experts had access to all relevant information from the HUNT4 70+ dataset, such as cognitive tests, patient history, physical diseases including stroke, function in activities of daily living, neuropsychiatric symptoms assessment and a structured interview with the closest family proxy.

### Blood pressure, dementia status and covariates

BP (mmHg) was measured in HUNT1–4. In the study population 75% had BP measured at all waves (HUNT1–4), 90% had a BP measurement from at least 3 waves, 96% had a BP measurement from at least two waves, and 4% had a BP measurement from only one wave. Participants were included if there was at least one valid BP measurement in HUNT1–4 and a valid dementia assessment at HUNT4.

Dementia status was categorized as no dementia and dementia. Dementia subtypes were categorized as AD, VaD and “other dementia.” The category “other dementia” included LBD, FTD, mixed dementia, other specified dementia, and unspecified dementia.

Time dependent covariates at HUNT1–4 included self-reported antihypertensive medication use (yes/no), daily smoker (never, ever, current) and history of stroke (yes/no). Obesity was defined as body mass index [calculated as weight (in kilograms) divided by squared height (in meters)] ≥ 30 and included as a dichotomous time dependent variable. Time invariant covariates included the following: birth year, sex (male/female) and education level (compulsory, secondary, tertiary) obtained from the National Education Data Base (registry based data). Missing values for education (*n* = 25) were imputed as compulsory education. Missing values for history of stroke (*n* = 70) for HUNT2–4 were imputed based on reports on previous HUNT-study waves.

### Procedures for blood pressure assessment

At HUNT1 BP was assessed using a mercury sphygmomanometer, by trained nurses or technicians. BP was recorded twice in the seated position after resting for a minimum of 5 min (Gabin et al., 2017). In HUNT1 the mean of the first and the second readings was used to calculate mean systolic or diastolic BP.

In HUNT2-HUNT4, three repeated automated oscillometric BP-measurements were recorded at 1-min intervals. The measurements were started after the participant was seated for 2 min with the cuff on the arm, and the arm resting steadily on a table. The mean of the second and third readings were used to calculate mean systolic or diastolic BP.

In HUNT2, measurements were done at the stationary assessment team in the five larger municipalities by oscillometry., using a Critikon Dinamap monitor (845XT and XL9301) (Gabin et al., 2017).

In the HUNT3 study, BP and heart rate were measured using a Critikon Dinamap (8,100) based on oscillometry (Krokstad et al., 2013). Dinamap XL model 9301 (Johnson & Johnson Medical Inc.) was also used for the measurements by the mobile team in the 19 smaller municipalities in both HUNT2 and HUNT3. The Dinamap XL model 9301 measures mean arterial pressure directly, and hence does not estimate it from systolic and diastolic pressure. In HUNT4 the Dinamap CARESCAPE V100 (GE Healthcare) with GE TruSignal for pulse oximetry was used, also based on automatic oscillometry.

### Cognitive assessments

In HUNT4 70+ the following cognitive assessment instruments were applied:

The Montreal Cognitive Assessment (MoCA) scale is a multidomain cognitive screening instrument that tests memory, visuospatial and executive functions, naming, attention, abstraction, language, and orientation. Scores range from 0 to 30; higher scores indicate better cognitive function (Nasreddine et al., 2005).

The Word list from the Consortium to Establish a Registry for Alzheimer’s Diseases (CERAD) (ten-word immediate and delayed memory test) tests memory with a list of 10 words that the person being tested is asked to recall after each of three initial presentations (immediate recall) (score 0–30) and again after 10 min (delayed recall) (score 0–10) (Morris et al., 1989). In nursing home patients with moderate to severe dementia, the Severe Impairment Battery 8-item version was applied (Schmitt et al., 2013).

### Ethics

This study was approved by the Regional Committee for Medical and Health Research Ethics in Norway (REK Southeast 251687) and the Norwegian Center for Research Data (NSD 571736). Participation in the HUNT studies was based on an informed written consent.

### Statistical methods

Stata 16 was used for all analyses. SBP was used as the outcome in a random intercept and random slope multilevel mixed-effects linear regression model with HUNT study survey as time variable (1–4) and dementia status at HUNT4 as independent variable. Year of birth, sex, and educational level were added to the model as time invariant adjustment variables, while BP medication use, obesity, daily smoking, and history of stroke were allowed to vary over the HUNT surveys 1–4 and treated as time dependent covariates in the regression model. All interactions between age, sex and dementia status were included. SBP values by dementia status, sex and survey time point were predicted from the regression model *post hoc* using the margins command. Stratified predictions were performed to investigate differences between men and women, between age groups (birth years 1914–34 vs. 1935–49), and between dementia subtypes. Analyses were run on the total study population (*n* = 9720) in a minimally adjusted model with these variables included: dementia, time, time*dementia, birth year, sex, as well as in the study population with non-missing values for all the adjustment variables (*n* = 9484).

In a second multilevel mixed-effects linear regression model analysis (random intercept and random slope), age was used as the time variable, thus the age trajectories by dementia status at HUNT4 70+ were modeled. Age was included as a linear and quadratic term and the interactions with dementia status were included. The model was adjusted by all the adjustment variables mentioned above and performed on the sample with non-missing values for all the adjustment variables (*n* = 9484).

Since only two BP measurements were performed in HUNT1 and three BP measurements were taken in HUNT2–4, we did three sensitivity analyses using the same SBP measurement at all surveys; first we used the first SBP measurement at all surveys, secondly, we used the second measurement at all surveys, and lastly we used the mean from reading number one and two for all surveys.

## Results

At HUNT4 the mean age was 77.6 years (*SD* 6.0) (range 70–102) and 54% were women. In 1984, the initial year of the HUNT study (HUNT1), the mean age was 44.1 years (*SD* 6.4) (range 35–70). During cognitive assessments at HUNT4, 1503 (15.5%) were found to have dementia, of which 856 (8.8%) had AD, 156 (1.6%) had VaD and 491 (5.1%) had other types of dementia. In the total sample, mean SBP increased from 129.2 (*SD* 15.9) mmHg in HUNT1 to 138.8 (*SD* 19.2) mmHg in HUNT2 and remained stable from HUNT2 to HUNT3 (137.6 mmHg, *SD* 18.9) and HUNT4 (139.4 mmHg, *SD* 20.2). Descriptive characteristics of the population from HUNT1 to HUNT4, by dementia status at HUNT4 are presented in Table 1.

**TABLE 1:** Characteristics of the study participants at the HUNT surveys (HUNT1–4: 1984, 1995, 2006, 2017) by dementia status (assessed at HUNT4).

Characteristic	No dementia at HUNT4				Dementia at HUNT4			
	HUNT1	HUNT2	HUNT3	HUNT4	HUNT1	HUNT2	HUNT3	HUNT4
	1984	1995	2006	2017	1984	1995	2006	2017
No. of participants with BP measurements	7,295	7,267	7,214	8,181	1,358	1,316	1,138	1,403
Age mean (SD), years	43.7 (5.7)	55.3 (5.7)	66.4 (5.6)	76.8 (5.5)	50.5 (7.5)	62.1 (7.5)	72.6 (7.1)	82.5 (7.2)
Women (%)	3,993 (54.7)	3,976 (54.7)	3,904 (54.1)	4,373 (53.4)	814 (59.9)	794 (60.3)	673 (59.1)	809 (57.7)
Men (%)	3,302 (45.3)	3,291 (45.3)	3,309 (45.9)	3,808 (46.6)	544 (40.1)	522 (39.7)	465 (40.9)	594 (42.3)
Education (%)								
Compulsory	1,670 (22.9)	1,634 (22.5)	1,565 (21.7)	1,835 (22.4)	610 (44.9)	581 (44.1)	481 (42.3)	613 (43.7)
Secondary	3,200 (43.9)	3,169 (43.6)	3,142 (43.5)	3,475 (42.5)	517 (38.1)	509 (38.7)	434 (38.1)	535 (38.1)
Tertiary	2,425 (33.2)	2,464 (33.9)	2,507 (34.7)	2,871 (35.1)	231 (17)	226 (17.2)	223 (19.6)	255 (18.2)
Systolic BP mean (SD) mm Hg	128.2 (15.2)	137.5 (18.4)	137.1 (18.5)	140.1 (19.9)	134.7 (18.2)	146.1 (21.7)	140.8 (20.7)	134.9 (21.6)
Antihypertensive use (%)	301 (4.1)	843 (11.6)	2,519 (34.8)	4,130 (65.7)	125 (9.1)	285 (21.6)	554 (48.4)	538 (65.2)
Smoking status (%)								
Current	1,595 (25.2)	1,507 (20.9)	814 (11.6)	493 (6.2)	313 (26.5)	269 (20.7)	121 (11.1)	71 (6.6)
Previous	1,837 (29.0)	2,497 (34.6)	2,971 (42.2)	4,025 (50.5)	303 (25.6)	430 (33.1)	432 (39.9)	536 (50.0)
Never	2,904 (45.9)	3,209 (44.5)	3,248 (46.2)	3,457 (43.4)	567 (47.9)	601 (46.2)	529 (48.9)	466 (43.4)
Obesity (BMI > 30) (%)	438 (6.0)	1,139 (15.7)	1,765 (24.4)	1,696 (21.4)	132 (9.7)	274 (20.9)	304 (26.8)	396 (29.9)
History of stroke (%)	8 (0.1)	49 (0.7)	254 (3.5)	653 (8.8)	7 (0.5)	21 (1.6)	92 (8.0)	226 (23.2)

BMI, body mass index; BP, blood pressure; HUNT, The Trøndelag Health Study. Missing N in antihypertensive use: 1,007, 1,132, 1,330, 2,607 in HUNT1,2,3,4, respectively; missing N in smoking status: 2,198, 1,207, 1,605, 672 in HUNT1,2,3,4, respectively; missing N in obesity: 1,070, 1,148, 1,362, 473 in HUNT1,2,3,4, respectively; missing N in history of stroke: 1,008, 1,131, 1,319, 1,363 in HUNT1,2,3,4, respectively.

### Systolic blood pressure trajectories by all-cause dementia status at HUNT4, adjusted for confounders

**FIGURE 1:**
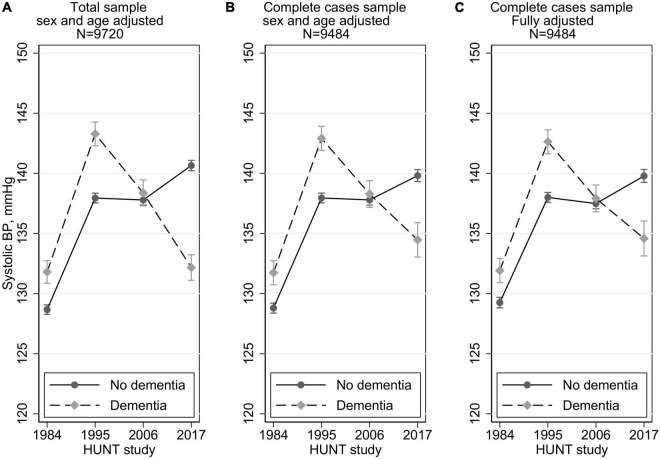
Systolic blood pressure trajectories by dementia status (yes/no) at HUNT4 70+ in 2017–19 with 95% confidence intervals. Multilevel mixed methods with random intercept and slope. In the fully adjusted models in C, these time-dependent variables are included: blood pressure medication (yes/no), smoking (current, previous, never), obesity (yes/no), and history of stroke (yes/no), and the following time-invariant variables: education (compulsory, secondary, tertiary), birth year, and sex.

SBP from HUNT1 to HUNT4 comparing those with and without dementia is presented in (Figure 1A) total sample adjusted for sex and age, (Figure 1B) complete cases sample adjusted for sex and age and (Figure 1C) complete cases fully adjusted for sex, age, antihypertensive use, obesity, smoking status and history of stroke.

Adjusted by sex and birth year (centered at 1939, and 45% men), the SBP trajectory for those with dementia, for HUNT study waves 1–4 was 131.8, 143.3, 138.4, 132.2 mmHg, respectively. For those without dementia the SBP trajectory was 128.7, 137.9, 137.8, 140.7 mmHg from HUNT1–4 (Figure 1A). Thus, compared to those without dementia, those with dementia at HUNT4, had higher SBP levels at both HUNT1 (3.1 mmHg higher, 95%CI 2.1, 4.2) and at HUNT2 (5.3 mmHg higher, 95%CI 4.3, 6.4) (Table 2). At HUNT3 the SBP levels were similar (0.6 mmHg higher, 95%CI –0.6, 1.8), and at HUNT4 the pattern was reversed; the BP level was 8.5 mmHg lower (95%CI –9.6, –7.3) in the dementia group. In the sample with no missing values for all confounders (*n* = 9,484), the SBP trajectories by dementia status at HUNT4 were similar to the full sample (*N* = 9,720), except that there was a slightly smaller difference at HUNT4; the level was 5.3 mmHg lower (95%CI –6.9, –3.8) in the dementia group (Figure 1B and Table 2). In a fully adjusted model, with education, antihypertensive medication, smoking, obesity and stroke, results were minimally attenuated and almost identical to those adjusted only for age and birth year (Figure 1C and Table 2).

**TABLE 2:** Absolute difference in SBP (mmHg) at HUNT1, HUNT2, HUNT3 and HUNT4, respectively, for those with dementia at HUNT4 vs those without dementia at HUNT4 (95% CI).

	Number of participants (%)	HUNT1 1984	HUNT2 1995	HUNT3 2006	HUNT4 2017
**All**					
Model 1	9,720	3.1 (2.1, 4.2)	5.3 (4.3, 6.4)	0.6 (–0.6, 1.8)	–8.5 (–9.6, –7.3)
Model 2	9,484	2.9 (1.8, 4.1)	5.0 (3.9, 6.0)	0.5 (–0.7, 1.7)	–5.3 (–6.9, –3.8)
Model 3	9,484	2.7 (1.6, 3.7)	4.6 (3.6, 5.7)	0.4 (–0.8, 1.6)	–5.2 (–6.7, –3.7)
**By sex (A), model 3**					
Men	4,313 (45.5)	0.9 (–0.7, 2.6)	2.4 (0.7, 4.0)	–0.2 (–2.0, 1.6)	–5.0 (–7.3, –2.8)
Women	5,171 (54.5)	4.2 (2.8, 5.6)	6.2 (4.8, 7.6)	0.8 (–0.8, 2.3)	–5.6 (–7.6, –3.5)
**By birth cohort (B), model 3**					
Born 1935 or later	7,247 (76.4)	2.5 (0.9, 4.0)	4.5 (3.0, 6.1)	2.1 (0.4, 3.8)	–2.1 (–4.1, –0.01)
Born before 1935	2,237 (23.6)	4.3 (2.8, 5.9)	4.8 (3.2, 6.4)	1.4 (–0.4, 3.1)	–4.4 (–6.8, –2.0)
**Dementia type (C), model 3**					
No dementia	8,037 (84.7)	–	–	–	–
AD	831 (8.8)	2.1 (0.8, 3.5)	3.4 (2.1, 4.8)	–0.6 (–2.1, 0.9)	–5.7 (–7.7, –3.7)
VaD	147 (1.5)	4.6 (1.6, 7.6)	9.9 (6.9, 12.9)	6.0 (2.6, 9.3)	–2.1 (–6.2, 2.0)
Other dementias	469 (4.9)	3.0 (1.3, 4.8)	5.2 (3.4, 7.0)	0.6 (–1.4, 2.6)	–5.2 (–7.8, –2.6)

Model 1 Total sample (*N* = 9,720), age and sex adjusted; Model 2 Complete cases sample (*N* = 9,484), age and sex adjusted; Model 3 Complete cases sample (*N* = 9,484), adjusted by age, sex, education, and histories of blood pressure medication, smoking, obesity, and stroke.

### Systolic blood pressure trajectories by dementia status at HUNT4: Differences according to sex, birth cohort and dementia subtype (fully adjusted model, complete case sample *n* = 9484)

**FIGURE 2:**
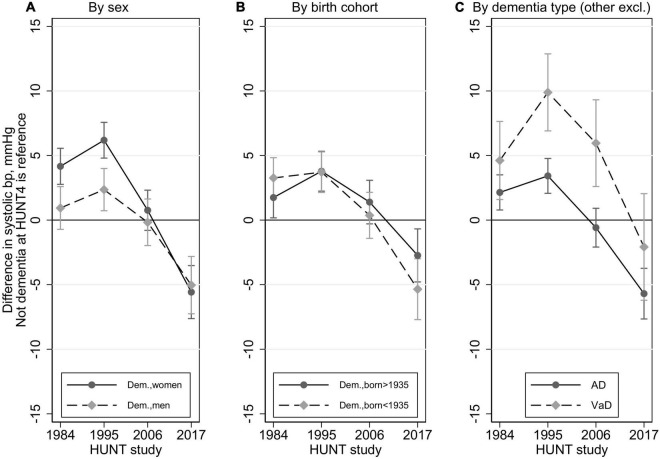
Trajectories in difference in systolic blood pressure between those with dementia at HUNT4 70+ (2017–19) vs those without dementia (reference line 0) by sex, birth cohort, and dementia type (restricted to AD and VaD) with 95% confidence intervals. Multilevel mixed methods with random intercept and slope. In all models, these time-dependent variables are included: blood pressure medication (yes/no), smoking (current, previous, never), obesity (yes/no), and history of stroke (yes/no), and the following time invariant variables: education (compulsory, secondary, tertiary), birth year, and sex.

The SBP difference between the no dementia group and the dementia group at HUNT4, over the four HUNT waves is shown in Figure 2 and Table 2, with stratification by (Figure 2A) sex, (Figure 2B) birth cohort and (Figure 2C) type of dementia.

Compared with the no dementia group, women in the dementia group had a higher SBP at HUNT1 and HUNT2 and a lower SBP at HUNT4. There was no difference between the groups at HUNT3. In men, SBP was higher in the dementia group at HUNT2 and lower in the dementia group at HUNT4, whereas there was no difference between the groups at HUNT1 and HUNT3. The difference in SBP according to dementia status at HUNT4, was significantly larger in women compared to that in men at HUNT1 (*p* = 0.003) and HUNT2 (*p* < 0.001), while the difference was similar across sexes at HUNT3 (*p* = 0.43) and HUNT4 (*p* = 0.72) (Figure 2A and Table 2).

The difference in SBP according to dementia status at HUNT4 was similar across birth cohorts (Figure 2B and Table 2).

For those without dementia the SBP trajectory was 129.1, 137.8, 137.3, 139.7 mmHg from HUNT1–4 (almost identical to the trajectories described above for the full sample). For those with AD the SBP trajectory was 131.2, 141.3, 136.8, 134.0 mmHg from HUNT1–4, and for those with VaD the SBP trajectory was 133.7, 147.7, 143.3, 137.6 mmHg from HUNT1–4. Thus, there were substantial differences among dementia subtypes. Compared to the no dementia group, the VaD group had a higher SBP at HUNT1, HUNT2 and HUNT3, but there was no difference at HUNT4. Compared to the no dementia group, the AD group had a higher SBP at HUNT1, HUNT2 and a lower SBP at HUNT4. There was no difference at HUNT3. Those with VaD had significantly higher SBP than those with AD, both at HUNT2 (6.5 mmHg higher, *p* < 0.001) and HUNT3 (6.5 mmHg higher, *p* < 0.001) (Figure 2C and Table 2). The VaD group had similar SBP as the AD group at HUNT1 (*p* = 0.14) and HUNT4 (*p* = 0.12).

The SBP of the “other dementias” group did not differ from the AD group from HUNT1-HUNT4 (Table 2).

### Age trajectories

**FIGURE 3:**
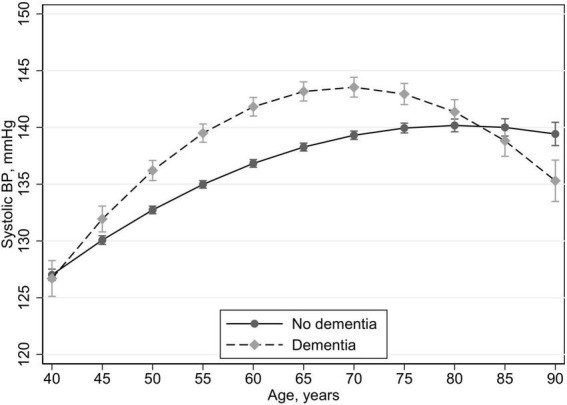
Age trajectories in systolic blood pressure between those with dementia at HUNT4 70+ (2017–19) vs those without dementia with 95% confidence intervals. Multilevel mixed methods with random intercept. In all models, these time-dependent variables are included: blood pressure medication (yes/no), smoking (current, previous, never), obesity (yes/no), and history of stroke (yes/no), and the following time invariant variables: education (compulsory, secondary, tertiary), and sex.

Fully adjusted age trajectories in SBP between those with and without dementia at HUNT4 70+ are presented in Figure 3. By visual inspection we saw in the dementia group a steady increase in SBP until 65 years of age, a stable level from 65 to 70 and a steady decrease from 70 to 90 years of age. In the no dementia group we saw that SBP increased until age 80 before it leveled off from 80 to 90.

Compared to the no dementia group the dementia group had higher SBP from 45 to 75 years of age. At 80 and 85 years of age there was no difference between the groups and at 90 years of age the dementia group had lower SBP than the no dementia group.

### Sensitivity analyses

Using alternative BP measurements in the analyses did not impact the main findings (results not shown). Using the first BP measurement across all HUNT surveys 1–4, instead of the mean of two readings, shifted the BP slightly upwards for all groups. Thus, the choice of BP measure was robust and did not affect our conclusions.

## Discussion

In this retrospective population-based cohort study, we found that persons with dementia had a higher SBP in HUNT1-HUNT2 but a lower SBP in HUNT4. The decrease in SBP in the dementia group started 10–20 years prior to dementia diagnosis at HUNT4. The results were largely confirmed in both the AD and VaD groups. Although the overall pattern of SBP trajectories remained the same across sex and age groups, some differences between groups were observed.

A pattern with higher BP in midlife but lower BP in late life in persons with dementia, compared to persons without dementia was demonstrated in the seminal study by Skoog et al. (1996) and has later been confirmed in several studies (Qiu et al., 2005; Li et al., 2007; Abell et al., 2018). However, studies on midlife hypertension cover a wide age range. Also in the present study, the age range of the participants at each assessment spans more than 30 years. However, when considering the sample in two age groups (35–49 and 50–70 years at HUNT1), we found that the pattern with higher SBP at HUNT1 and HUNT2 and lower SBP at HUNT4, in those with dementia at HUNT4 remained the same in both age groups. When modeling age trajectories of SBP we found substantial differences between the groups. The dementia group had an increase in SBP until the age of 70 and a significant drop in SBP afterward. The no dementia group experienced a slower increase in SBP but no significant drop in SBP in old age. These findings indicate that higher SBP is associated with an increased risk for dementia over a long period, from 45 years until 75 years of age and that it is only in later life that lower SBP is associated with a higher risk of dementia. These findings highlight the importance of monitoring SBP closely in older people with dementia as antihypertensive medication might need to be adjusted or even deprescribed to avoid negative outcomes, such as increased morbidity and mortality (Benetos et al., 2015). Furthermore, current evidence does not show any clear benefit of initiation of antihypertensive treatment in older age groups (Benetos et al., 2019).

Potential mechanisms that may explain the association between hypertension and dementia risk are multifaceted. It seems that higher BP and older age have a synergistic deleterious effect on the structural and functional integrity of the cerebral microcirculation (Ungvari et al., 2021). Hypertension leads to dysregulation of cerebral blood flow. This in turn exposes the cerebral microvessels to hemodynamic instability causing pathological changes termed small vessel disease. This includes endothelial dysfunction, lipohyalinosis, fibrinoid necrosis, lacunes and microhaemorrhages (McGrath et al., 2017). All these events are associated with cognitive decline. Chronic hypertension may also disrupt blood-brain-barrier (BBB) function impeding transport of essential substances into the brain and transport of waste products out of the brain. Furthermore, BBB disruption promotes neuroinflammation, synaptic dysfunction and myelin damage contributing to cognitive decline and dementia and exacerbate amyloid pathologies associated with Alzheimer’s disease (Ungvari et al., 2021). Chronic hypertension is also associated with arteriosclerosis and cardiac failure, both conditions which may negatively affect cerebral blood flow and thereby cause cognitive decline. Long-standing hypertension may induce a state of hypoperfusion in the brain. It has been postulated that brain hypoperfusion is involved in the pathogenesis of AD, by constituting an upstream event before amyloid formation (De la Torre, 2018).

There might be several explanations as to why midlife hypertension, but not late-life hypertension is associated with dementia. Hypertension usually begins long before the neurodegenerative process and it acts as a powerful contributor to cognitive impairment in midlife (Veldsman et al., 2020). While hypertension is one of very few contributors to cognitive impairment in midlife, its contribution might be obscured by several other factors in late life, such as neurodegeneration because of AD. Other factors associated with development of dementia, such as people with dementia becoming more immobile, more fragile and being underweight, might in combination contribute to a decrease in blood pressure along the disease course. Recently, a new evolutionary interpretation of the brain’s circulation has been proposed, indicating that the brain circulation comprises complementary low-pressure and high-pressure systems (the ambibaric brain). This model highlights the need for the development of methods of assessing the best blood pressure for the individual brains and for close monitoring of BP to optimize brain health (Hachinski and Østergaard, 2021).

Most previous studies report that dementia and AD are more common in women than in men whereas VaD is more common in men (Cao et al., 2020). This was also found in our recent study using the same HUNT4 70+ group as the present study (Gjøra et al., 2021). Suggested explanations for this sex difference include longevity (women live longer than men), biological differences (hormones, epigenetics, frailty), differences in cognitive performance and gendered social roles and opportunities (Andrew and Tierney, 2018). Recent studies have indicated that sex differences in dementia prevalence may be influenced by sex differences in the profile of dementia risk factors. A recent review concluded that higher midlife SBP was associated with a greater risk of all-cause dementia, AD and VaD in women compared to men (Blanken and Nation, 2020). Another recent study found that mid-adulthood hypertension was associated with increased dementia risk in women, but not in men (Gilsanz et al., 2017). A large UK biobank study found that the association between several midlife cardiovascular risk factors and risk of dementia did not differ between the sexes, but BP affected men and women differently. The relationship between higher SBP and dementia risk was U-shaped in men but had a dose-response relationship in women. This difference was not affected by antihypertensive use and was consistent across dementia subtypes, like AD and VaD (Gong et al., 2021). Our study strengthens the idea that sex differences exist since SBP was higher at HUNT1 in the dementia group only in women. Furthermore, the difference in SBP between those with and without dementia was larger in women than in men at both HUNT1 and HUNT2. These differences remained when the analysis was adjusted for antihypertensive use and other risk factors which may differ between the sexes.

The association between midlife hypertension and AD risk is unclear, with most studies showing consistent association between increased midlife diastolic BP and AD risk, whereas studies regarding increased midlife BP and AD risk are conflicting. An association between late-life hypotension and AD risk has been documented in several studies (Walker et al., 2017). Our study confirms a clear pattern of SBP trajectory and AD risk, where a higher SBP in midlife and a lower SBP in late life was associated with a diagnosis of AD in late life.

The evidence supporting an association between midlife hypertension and increased VaD risk is stronger than for AD risk but the results regarding BP and VaD risk in old age are conflicting. Only a few studies have addressed this specifically (Walker et al., 2017). A registry-based study of 4.28 million individuals found that higher SBP was associated with increased VaD risk, irrespective of preceding transient ischemic attack or stroke but no inverse association in old age (Emdin et al., 2016). Our findings confirm that people with VaD have higher SBP in midlife than those with AD. Additionally we found that SBP decreases substantially in the VaD group with increasing age. However, not to the extent that VaD was associated with lower SBP compared to the no dementia group, as observed in the AD group at HUNT4. Comparisons between the AD and VaD group should be interpreted with caution as there probably is considerable overlap with a large group of people with mixed AD and VaD pathology (Custodio et al., 2017). One could argue that most dementias constitute a mix, often including AD pathology with a typical case harboring several pathologies (Boyle et al., 2018).

### Strengths and limitations

The main strength of the present study is the large population-based sample, a follow-up period of more than 30 years and a thorough consensus-based method for diagnosing dementia and subtypes of dementia. By including nursing-home patients, the sample covers all levels of dementia. Furthermore, BP was measured in a standardized way and 75% of the participants had BP measurements at all four assessments. We were able to adjust for several potential confounders, limiting the chance of residual confounding.

There are a few limitations that should be taken into consideration when interpreting the results. We did not have information about race/ethnicity. However, recent reports show that only 1.6% of the population 67 + in the catchment area of the present study had a minority background (StatBank Norway, 2022). Hence, the present results may not be generalized to groups beyond a Nordic population. Although the diagnostic process conforms to the quality indicators of population-based dementia studies, the reliance on data collected by others and the lack of biomarker data may make the diagnosis less valid, especially regarding subtypes of dementia. The lack of cognitive assessments prior to HUNT4 is a major limitation. It is reasonable to assume that the participants did not have dementia at HUNT1 and HUNT2 and that very few if any had dementia at HUNT3. However, it is difficult to gauge the importance of reverse causality, especially because of disease development in the preclinical stage in some of the dementia subtypes. Even though the participation rate is relatively high, some selection bias is likely. A study on non-participation in HUNT3 showed that those who did not participate had lower socioeconomic status, higher mortality and a higher prevalence of chronic diseases (Krokstad et al., 2013). Another limitation is the substantial amount of self-reported data, which is inherent to most large-scale population studies. Even though we have adjusted for the most common confounders residual confounding cannot be excluded. Finally, our study may be prone to survival bias. This would most likely attenuate the association between the exposure in midlife (BP) and the outcome (dementia).

## Conclusion

In the present study, dementia was associated with systolic hypertension in midlife but not in late life. In general, the same pattern existed across sex, age groups and types of dementia although some variation was apparent. Our findings underline that the trajectories of blood pressure should be closely monitored in clinical practice and the need for continuing antihypertensive treatment should be reappraised regularly. Separate blood pressure targets for men and women may need to be developed. To identify the ideal blood pressure for the individual brain is an urgent question in personalized medicine. Future studies should also include measurements of standing and sitting/lying blood pressure since orthostatic instability may contribute to the cognitive impairment.

## Data availability statement

The raw data supporting the conclusions of this article will be made available by the authors, without undue reservation.

## Ethics statement

The studies involving human participants were reviewed and approved by the Regional Committee for Medical and Health Research Ethics in Norway. The patients/participants provided their written informed consent to participate in this study.

## Author contributions

GS, JS, CT, and BS were responsible for the conception and the design of the study. JS prepared the dataset. BS, GS, YW, and JS contributed to the analysis of data. GS and BS wrote the first draft. All authors gave input to the analysis plan, contributed to data interpretation and critical revisions of the manuscript, read, and approved the final manuscript.
